# Supplementary material for: Cognitively unimpaired adults’ reactions to disclosure of amyloid PET scan results
Source: PLoS One. 2020 Feb 13;15(2):e0229137. doi: 10.1371/journal.pone.0229137 (PMC7018056; doi:10.1371/journal.pone.0229137)
Supplement: S1 Interview guide — (DOCX) [file pone.0229137.s001.docx]

For the third part of the interview, I want to ask some questions about whether you have talked to other people about your amyloid PET scan result.

[The point of these questions is to elicit names of people they are close to/spend time with, and get a general idea about their social context. Once you feel you have gathered this, move on]

**Move to chart F: Usual practice.**

**Yes – Move to chart D: Results discussions**

No - I’d like to talk a little bit about why you haven’t discussed your amyloid PET scan result with anyone.

Thinking about the people you’ve just mentioned, have you talked to any of them about your amyloid PET scan result? Or did you tell anyone we haven’t discussed?

So it sounds like you:

- **told [PERSON/PEOPLE] your result because [REASON]**
- **decided not to tell [PERSON/PEOPLE] your result because [REASON]**
- **concealed your participation/result from [PERSON/PEOPLE] because [REASON]**

Does that sound about right?

**[Use judgment, may want to confirm after each bullet point summary]**

Is there anything you want to add to that?

**Move to Chart J**

Repeat shaded question for:

-Physical activity

-Prescription medications, vitamins, or herbal supplements

-Stress reduction

-Mental activities

-Other

**If the participant asks about what they *should* be doing:** Our goal in this study isn’t to suggest any particular thing you should be doing, just to find out what you *are* doing. **If they ask for AD/dementia prevention tips:** Advice on disease prevention is outside the scope of this study. You can speak with your doctor, or contact the NIH Alzheimer's Disease Education and Referral (ADEAR) Center ([www.nia.nih.gov/alzheimers or 1-800-438-4380](http://www.nia.nih.gov/alzheimers%20or%201-800-438-4380))

**Demographics**

**[If demographics already collected, skip to Closing – try to collect demographics when scheduling call if at all possible]**

Thank you so much for answering my questions. We’re almost done. Finally, I’d like to get just a little more information about you

- *[demographic questions]*
- Thanks for going through those with me.

**Closing**

That was my last question. Do you have any questions for me?

Thank you very much for talking with me today. We are sending you a $20 gift card to show our appreciation for your time today, and we will be calling you again in about a year. If you have any questions or concerns before then, please feel free to contact us.

What is the best way to reach you to schedule the follow up call? Is this number OK?
