# Supplementary material for: Cognitively unimpaired adults’ reactions to disclosure of amyloid PET scan results
Source: PLoS One. 2020 Feb 13;15(2):e0229137. doi: 10.1371/journal.pone.0229137 (PMC7018056; doi:10.1371/journal.pone.0229137)
Supplement: S2 Interview guide — (DOCX) [file pone.0229137.s002.docx]

**If participant told:**

**>5 people, move to Chart C**

**≤5 people, move to Chart D**

Note: Continue probing if participant mentions result unexpectedly shared with someone (i.e. by someone else or otherwise unplanned)

**Skip to Chart H if participant did not indicate any “not sharing” individuals in Interview 1**

**Skip to Chart J if participant did not include “thinking about sharing” individuals in interview 1**

**Skip to Chart L if participant did not include “concealing” individuals in interview 1**

**Sharing Summary**

Next I want to review a few points to make sure I have an OK understanding of what you’ve told me…

- Overall summary of points related to stigma (based on protocol core questions)
- Ask for clarification about any unclear sections
- Ask if participant has anything to add

Repeat shaded question for:

-Physical activity

-Prescription medications, vitamins, or herbal supplements

-Stress reduction

-Mental activities

-Other

**If the participant asks about what they *should* be doing:** Our goal in this study isn’t to suggest any particular thing you should be doing, just to find out what you *are* doing. **If they ask for AD/dementia prevention tips:** Advice on disease prevention is outside the scope of this study. You can speak with your doctor, or contact the NIH Alzheimer's Disease Education and Referral (ADEAR) Center ([www.nia.nih.gov/alzheimers or 1-800-438-4380](http://www.nia.nih.gov/alzheimers%20or%201-800-438-4380))

**Closing**

That was my last question. Do you have any questions for me?

Thank you very much for talking with me. We are sending you a $20 gift card to show our appreciation for your time today.

**Permission to Contact**

Currently this is the final interview scheduled with you for the SOKRATES study. However, it is possible that we will want to interview you again in the future. Is it OK for us to get in touch with you if we want to interview you again later? There is no obligation to complete any future interviews; you are just granting permission to contact you again.
