# Supplementary material for: Cognitively unimpaired adults’ reactions to disclosure of amyloid PET scan results
Source: PLoS One. 2020 Feb 13;15(2):e0229137. doi: 10.1371/journal.pone.0229137 (PMC7018056; doi:10.1371/journal.pone.0229137)
Supplement: Attachment — Submitted filename: Response to Reviewers.docx [file pone.0229137.s003.docx]

**Reviewer #1:**

Submitted article does not identify/clarify whether or not intended for special issue on "reward and decision making", so general submission to PLOS ONE assumed without consideration for special issue.

- We apologize for any confusion; the cover letter noted that this paper was submitted to the “Early Diagnosis and Treatment of Alzheimer’s Disease” Call for Papers.

Title: "Cognitively Unimpaired Adults’ Reactions to Amyloid Disclosure." There is a problem with use of the phrase "amyloid disclosure" in the title. Why not use the full phrase "amyloid imaging results disclosure"? What is the meaning of the shortened phrase "amyloid disclosure"? Does it refer to which testing or imaging of which kind of amyloid? Is it possible to "disclose amyloid"? Or is that a non-sensical phrase for which example precedents do not exist in published medical science? Certainly, we do not publish medical scientific reports on "blood disclosure" or "bone disclosure" or any other kind of bio-tissue or bio-molecule!!!

- We have changed the title to: Cognitively Unimpaired Adults’ Reactions to Disclosure of Amyloid PET Scan Results.

Abstract contains other shortened phrases such as "elevated participants" and "not-elevated participants" that also result in potentially confusing and/or non-sensical abuses of English language. Again, this reviewer objects to these misuses of the English language. Presumably, the research participants are neither elevated nor not-elevated! Rather, their amyloid imaging results have been interpreted as elevated or not-elevated.

- We note that Reviewer 2 also shared this concern about use of “elevated” and “not-elevated.” We have made responsive edits throughout the manuscript to reflect that we are discussing “elevated” and “not-elevated” amyloid PET scan result.

Abstract contains the phrase "urgent need to understand the effects of biomarker disclosure on this population" which raises the question: what is the "urgency"? In clinical medical contexts, use of the words "emergent" or "urgent" do have relevant and important meanings. However, majority of evidence published so far on amyloid imaging results disclosure suggests that the risks if any are minimal, and certainly do not rise to the level or either emergent or urgent. So what is the rationale or justification for use of the word "urgent"? Why over-dramatize and raise an alarm with the word "urgent" if minimal if any harm will occur? And if the authors do believe that harm will occur then they should provide the explanation, rationale and evidence for the harm that they fear will occur with probability estimates for the numbers of potential patients impacted. How do these predicted numbers compare to the numbers of persons involved in the current opiates drug overdose epidemic plaguing America today? And what constitutes an emergency or urgency? The authors should be careful before using language that creates drama frightening people unnecessarily.

- We have deleted the word “urgent” from the abstract as it was not our intention to unnecessarily frighten anyone.

Data availability reported as "fully available without restriction" with additional statement that "All relevant data are within the manuscript and its Supporting Information files." However, this reviewer did not find any relevant raw data in either the manuscript or supporting information. There are no tables or URL links to repositories of raw data found anywhere in the manuscript or supporting information files. The manuscript contains only tables of analyzed results.

- This was in error. The transcripts are not fully available without restriction as they contain identifiable information.

Introduction contains the statement "The extant empirical literature on such disclosure is extremely limited.(11),(12)" However, this statement is neither correct nor current and up-to-date. Despite presence of bibliography with a total of 30 references, some important literature has been omitted. If the total number of references must be constrained, then would not some of these missing references be more important than some of those that were mentioned? Criteria for inclusion of literature citations should be relevance to discussion and argument of issues and claims in the submitted manuscript.

- Reviewer 1’s point that there is a literature on AD biomarker disclosure is well taken. We have revised the sentence as follows: “The extant empirical literature on disclosure of amyloid PET scan results to cognitive unimpaired adults is, however, limited.”

Missing literature:

Book chapter associated with talk and slide presentation at AAIC 2019 Los Angeles on Fri Jul 12 at 11:30 AM. "Disclosure of Amyloid Imaging Results" 2019 by Jennifer H. Lingler (<https://www.alz.org/aaic/program/education-workshop-neuroimaging.asp)>. This work important as a recent comprehensive survey that should be up to date for amyloid imaging results disclosure studies as of the AAIC conference in July 2019; it appears that none of the papers by Lingler, neither recent nor past, have been discussed by the authors of the manuscript submitted to PLOS ONE.

- We have added a citation to Lingler & Klunk (2013) as that paper specifically recommends against disclosure of amyloid imaging results to cognitively normal research participants.
- We have also added a citation to Kim and Lingler (2019).

"Safety of Disclosing Amyloid Imaging Results to MCI and AD Patients" 2018 Taswell et al (<http://mhfmjournal.com/pdf/MHFM-120.pdf)>. This work important as the largest size study to date of amyloid imaging results disclosure, also important for the careful use of robust statistical methods in the data analysis. This important clinical trial has not been cited and discussed by the authors of the manuscript submitted to PLOS ONE.

- We have added a citation to Taswell et al (2018). We note, however, that this study looked at disclosure of results to adults with MCI and dementia rather than to cognitively unimpaired adults.

Methods contains the statement "Psychological measures of well-being are collected for all A4 and LEARN participants and are not reported here." Why not?!? What is the rationale and justification for NOT reporting and analyzing the results from the psychological measures of well-being for the research participants? If the data are available and analyzed correctly, then they should be reported.

- The psychological measures are proprietary to the A4 and LEARN studies and are not presently available for analysis or for publication by the SOKRATES team. We have deleted this sentence as unnecessary.

Results contain the statement "No notable differences in results were observed by age or gender." However, there are no group-wise results based on age group based analysis presented in the manuscript with evidence to support this claim which contradicts intuition and common life experience. The authors should either present the data and results to substantiate their claims, or at least, offer a hypothetical explanation for their failure to find differences between age groups. What about younger age groups in their 50s, 40s or 30s? This reviewer remains skeptical that there would be no differences between age groups. It's just a matter of clarifying the specific ages of the age groups over the life span.

- We have added a sentence explaining that Analyses included searching for differences among groups based on demographic variables.
- SOKRATES enrolled only participants aged 65 to 85 – because one eligibility criterion for the parent A4 and LEARN studies was being 65 to 85 years old. In our analyses, we looked at two age groups: 65 to 74 and 75+. We did not find differences by age between these two groups.
- It is an open question whether there would be differences by age if younger individuals received amyloid PET scan results. As that is not a question that can be answered with our present data set, we now note this as a direction for future research.

**Reviewer #2:**

Largent et al performed qualitative research to assess the reaction to a positive/negative amyloid PET result in cognitively normal individuals. This is an important topic as increasing numbers of cognitively healthy persons are undergoing amyloid PET for research and the return of study results is very much wanted by study participants a priori.

- We are glad that Reviewer 2 shares our belief that this is an important and timely topic for research and also the future of clinical care.

The number of participants is high, with a high proportion of persons (50 out of 80) with an elevated amyloid PET result. Overall the study appears to confirm that knowledge of an elevated amyloid PET may have negative consequences, mainly because of stigma associated with the disease.

- Thank you – we believe that the large sample size is a strength of our study.

On several occasions (e.g. p 6) the authors allude to the fact that the participants perceived memory problems beforehand. It would be of interest to better describe the cognitive complaints/status of the participants prior to disclosure: what was their motivation for enrolling in the study, did they fulfill criteria for subjective cognitive decline, was there a difference in test scores between those who had a positive versus a negative amyloid PET? If a person has subjective memory complaints, the elevated amyloid PET result may have a very different meaning than when no complaints existed beforehand and cognition was intact. The authors acknowledge in the Limitations section that an interview prior to disclosure could have resolved some of these questions.

- We have added additional information in the methods section to note that inclusion criteria for A4/LEARN required that participants were cognitively unimpaired. They scored within normal limits on baseline cognitive testing and were rated a zero on the CDR. Subjective cognitive complaints were permitted.
- Cognitive measures were collected in the A4 and LEARN studies but are not presently available for analysis or for publication by the SOKRATES team. The SOKRATES team did not collect cognitive measures. We agree with Reviewer 2 that this is a limitation and have noted it as such in our limitations section.

The disclosure process is described in a previous paper. This essential information may be summarized in a few sentences also in the current paper: which information was disclosed, e.g. in terms of positive and negative predictive value. By whom (physician, same researcher across all participants etc). Did the authors also use written information prior to or during the disclosure? It may be worthwile mentioning in the discussion that some of the reactions may be contingent on these factors.

- We thank Reviewer 2 for this suggestion and have added additional detail about the disclosure process on P4 of the manuscript.
- On page 17, we note that some of the reactions may be contingent on the standardized disclosure process.

I suggest the authors find a better term for ‘elevated participants’.

- We note that Reviewer 1 also shared this concern. We have made responsive edits throughout the manuscript to reflect that we are discussing “elevated” and “not-elevated” amyloid PET scan result.

An analogous qualitative research study was performed in amnestic MCI and disclosure of amyloid PET in a research context by Vanderschaeghe et al. Since the research method is similar and several elements in the participants’ reactions are similar, the authors may consider to refer to this study.

- We have added a citation to Vanderschaeghe et al (2017).

**Other:**

Please include a copy of the topic/interview guide used in the study, in both the original language and English, as Supporting Information, or include a citation if it has been published previously.

- As requested, we are including a copy of the interview guide used in the study.

Please provide additional details regarding participant consent. In the Methods section, please state why it was not possible to obtain written consent, how verbal consent was recorded and whether the ethics committee approved this consent procedure. If your study included minors, state whether you obtained consent from parents or guardians.

- We have updated the Methods section (PP 5-6) to provide additional details about consent. The IRB approved the waiver of written informed consent because the interviews were telephonic and the study was minimal risk.
- Our study did not include minors.
