# Supplementary material for: Cognitively unimpaired adults’ reactions to disclosure of amyloid PET scan results
Source: PLoS One. 2020 Feb 13;15(2):e0229137. doi: 10.1371/journal.pone.0229137 (PMC7018056; doi:10.1371/journal.pone.0229137)
Supplement: Attachment — Submitted filename: Response to Reviewers.docx [file pone.0229137.s004.docx]

**Reviewer #1:**

Limitations: Authors have not discussed, but should discuss, a major limitation of their study. They did not perform or report any quantitative psychometrics for comparisons of mood with after-disclosure versus before-disclosure comparisons evaluating individual change scores for participants as reported by Taswell et al in 2018 MHFM.

- In the limitations section, we now note that because SOKRATES is a qualitative study, we did not collect quantitative psychometrics. Such measures were, however, collected as part of A4, and will be published elsewhere (manuscript under review).
- Additionally, we have included a citation to Taswell et al (2018) here (Reference 25).

Authors also recommend that "Future studies should examine the effects of biomarker disclosure on study partners or pre-caregivers asked to monitor the cognition, function, and well-being of individuals with preclinical AD". But they did not cite the first publication of this general recommendation by Taswell et al in 2018 MHFM as follows: "we believe that a more productive area of possible future research would be evaluation of mood scale psychometrics for the patient’s primary caregiver instead of psychometrics for the patient". They should cite Taswell et al 2018 MHFM for the recommendation, and then discuss what they think might be differences between cognitively impaired patients and cognitively non-impaired volunteers and their respective families and study partner / caregivers.

- We have now noted that “Monitoring a cognitively unimpaired adult with elevated biomarkers is different work than being a caregiver for a person with dementia. Pre-caregivers reactions to biomarker results will likely differ from caregivers’ reactions because pre-caregivers are not performing the physical and emotional labor of caregiving, but instead, anticipating it.”
- Additionally, we have included a citation to Taswell et al (2018) here (Reference 25).

**Reviewer #2:**

(No Response)
